# THE IMPACTS OF WILDFIRES ON OLDER ADULTS: A SCOPING REVIEW

**DOI:** 10.1093/geroni/igac059.2307

**Published:** 2022-12-20

**Authors:** Carson De Fries, Colleen Melton, Rebecca Smith, Lisa Reyes Mason

**Affiliations:** University of Denver, Denver, Colorado, United States; University of Denver, Denver, Colorado, United States; University of Denver, Denver, Colorado, United States; University of Denver, Denver, Colorado, United States

## Abstract

Climate change is leading to worsening disasters that disproportionately impact certain populations, including older adults (Benevolenza & DeRigne, 2019). Older adults are more likely to encounter life-threatening challenges during disaster evacuation, less likely to receive disaster warnings, and more likely to experience greater financial losses following disasters (Acierno et al., 2006). While research has begun to measure these disparities, there is a gap in examining the effects of wildfire-specific disasters, which are increasing in intensity and severity (Hoover & Hanson, 2020). To examine this gap, scoping review methodology was used to analyze peer-reviewed studies of how wildfires affect older adults by looking at impacts and the disaster recovery cycle (i.e., preparedness, response, recovery, and mitigation). Authors screened 263 titles and abstracts, and 138 were reviewed in full. Eighty-one studies were included for data extraction and analysis. Preliminary findings illustrate that most literature focuses on the health impacts that older adults endure during and following wildfires, with a specific focus on the short and long-term effects of smoke and poor air quality on respiratory health. While previous literature has cited a need for community response strategies that incorporate the needs of older adults, few findings addressed firsthand experiences of older adults during a wildfire event. However, one unique finding was the incorporation of Aboriginal and Indigenous Elders’ knowledge into fire management strategies. While recommendations for incorporating the needs of older adults into policy planning were briefly mentioned, most articles focused on problem scope rather than evaluating potential solutions.

